# Structural characterization of life-extending *Caenorhabditis elegans* Lipid Binding Protein 8

**DOI:** 10.1038/s41598-019-46230-8

**Published:** 2019-07-10

**Authors:** Matthew C. Tillman, Manoj Khadka, Jonathon Duffy, Meng C. Wang, Eric A. Ortlund

**Affiliations:** 1 Department of Biochemistry, Emory University School of Medicine, 1510 Clifton Road, Atlanta, GA 30322 USA; 2 Huffington Center on Aging and Department of Molecular and Human Genetics, Baylor College of Medicine, Houston, Texas USA; 3 Howard Hughes Medical Institute, Baylor College of Medicine, Houston, TX 77030 USA; 4 Department of Developmental Biology, Baylor College of Medicine, Houston, TX USA

**Keywords:** X-ray crystallography, Fatty acids, Ageing, Intracellular signalling peptides and proteins

## Abstract

The lysosome plays a crucial role in the regulation of longevity. Lysosomal degradation is tightly coupled with autophagy that is induced by many longevity paradigms and required for lifespan extension. The lysosome also serves as a hub for signal transduction and regulates longevity via affecting nuclear transcription. One lysosome-to-nucleus retrograde signaling pathway is mediated by a lysosome-associated fatty acid binding protein LBP-8 in *Caenorhabditis elegans*. LBP-8 shuttles lysosomal lipids into the nucleus to activate lipid regulated nuclear receptors NHR-49 and NHR-80 and consequently promote longevity. However, the structural basis of LBP-8 action remains unclear. Here, we determined the first 1.3 Å high-resolution structure of this life-extending protein LBP-8, which allowed us to identify a structurally conserved nuclear localization signal and amino acids involved in lipid binding. Additionally, we described the range of fatty acids LBP-8 is capable of binding and show that it binds to life-extending ligands in worms such as oleic acid and oleoylethanolamide with high affinity.

## Introduction

Lysosomes are catabolically active cellular organelles and serve a vital role as the recycling center of the cell. Lysosomes contain various hydrolases, including proteases, lipases, nucleases, etc. that degrade damaged macromolecules and organelles in their highly acidic interior through a process termed autophagy^1^. As we age, we acquire various forms of damaged cellular macromolecules such as aggregated proteins, mutated DNA, and damaged organelles^2^. Given its significance in the clearance of these cellular damages, autophagy has been associated with a variety of longevity mechanisms. In the past few decades, molecular genetics studies in model organisms, including yeasts, worms, flies and mice, have demonstrated a series of lifespan-extending paradigms^2^. Interestingly, many of these paradigms induce autophagy, and the autophagy activity is required for their pro-longevity effects^3^. Thus, the lysosome can be linked with the longevity regulation through its involvement in the autophagic process.

On the other hand, the lysosome is not only the center for the degradation and recycling of cellular waste, but can also serve as the hub for organizing signal transduction and controlling nuclear transcription. With adequate amino acids, mTORC1 is recruited to the surface of the lysosome through its interaction with active Rag GTPases and Ragulator, and is then activated by the small GTPase Rheb^4^. The activation of mTORC1 can negatively regulate the nuclear translocation of TFEB, a master regulator of lysosome biogenesis, and affect lysosomal functions^4^. Both mTORC1 and TFEB have been implicated in the regulation of longevity^5–7^. More recently, Folick *et al.* reported a lysosome-to-nucleus retrograde lipid messenger signaling pathway in the regulation of longevity in *C elegans*. Upregulation of *LIPL-4*, a lysosomal lipid hydrolase extends lifespan through a process dependent upon the activation of nuclear receptors NHR-49 and NHR-80^8^. Both NHR-49, an orthologue of the peroxisome proliferator-activated receptors (PPARs) in vertebrates, and NHR-80, an orthologue of HNF4-α, bind to lipids and activate transcription responses crucial for the longevity regulation^9,10^. Folick *et al*. further identified a Lipid Binding Protein 8 (LBP-8) that mediates the retrograde signaling between lysosomal lipid hydrolysis and nuclear transcription. Upon the induction of LIPL-4, the *lbp-8* gene is transcriptionally up-regulated, and the LBP-8 protein is translocated into the nucleus from the lysosome. Interestingly, LBP-8 itself is also sufficient to prolong lifespan through activating NHR-49 and NHR-80^8^.

LBP-8 is a member of a larger family of proteins termed the intracellular lipid-binding proteins (iLBPs), which includes both fatty acid binding proteins (FABPs), cellular retinoic acid binding proteins (CRABPs), and cellular retinoid binding proteins (CRBPs). It is estimated that the iLBP family evolved in the animal kingdom over 1,000 MYA^11^. There are nine *C. elegans* FABPs, while humans have ten FABPs that are tissue specifically expressed. The human FABPs predominately bind to long-chained fatty acids, but some human FABPs bind larger hydrophobic molecules, such as bile acids, heme, and acyl-CoA^12–14^. They have been characterized to shuttle hydrophobic molecules to various cellular compartments, but of relevance here, certain human FABPs have been shown to shuttle nuclear receptor ligands into the nucleus to regulate nuclear receptor transcription^15–18^.

In this study, we characterized *C. elegans* LBP-8 using structural and biochemical techniques to further understand its function as a longevity promoting protein and to gain more insight into the family of iLBPs. We solved the structure of LBP-8 at 1.3 Å, which is the first structure of a *C. elegans* FABP, providing new insights into the diverse iLBP family. Additionally, we identified ligands that bind to LBP-8 in an unbiased manner using mass spectrometry (MS), supporting the role of LBP-8 as a shuttling protein for monounsaturated fatty acids and their derivatives.

## Results

### Overall structure of apo-LBP-8 and general comparison with other FABPs

**Figure 1:**
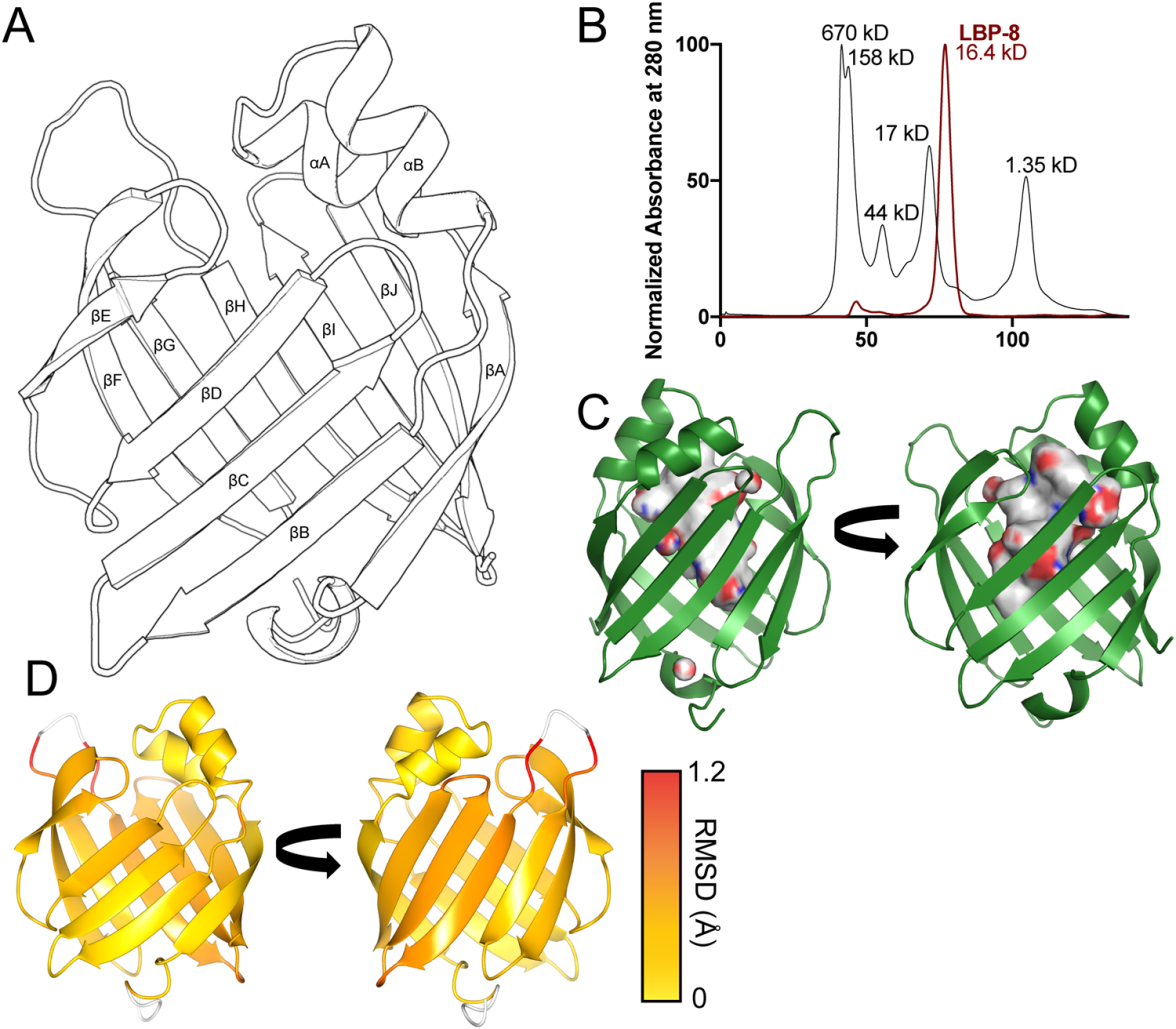
Structural overview of LBP-8. (**A**) Tertiary structure of apo-LBP-8. The protein adopts typical lipocalin fold; a beta barrel (βA- (βJ) capped with an alpha helical lid (αA- αB). (**B**) LBP-8 purifies as a monomer (16.4 kD). Size exclusion chromatography using HiLoad 16/60 Superdex 75 column comparing LBP-8 (red) and gel filtration standards (black). (**C**) Surface representation of the interior cavity of LBP-8. Nonpolar surface is colored grey, polar surfaces are colored red and blue (red indicates oxygen, blue indicates nitrogen). (**D**) ProSMART analysis conducted to determine r.m.s.d. between Cα backbone of LBP-8 and FABP4 bound to linoleic acid (PDB 2Q9S). Root mean square deviations (range: 0–1.2 Å) between structures were mapped onto LBP-8 structure with a color scale depicting low (yellow) to high (red) deviations. Unaligned regions are colored in white.

Overexpression of LBP-8 extends lifespan in worms, but the molecular mechanism explaining ligand binding or lysosome-nuclear lipid shuttling is not understood. To gain insight into these processes, we determined the first crystal structure of *C. elegans* LBP-8 (Fig. 1A). A crystal structure of heart FABP bound to stearic acid (PDB code 3WVM) was used as a search model to determine the initial phases since it shares the highest sequence similarity (37%) with LBP-8 of known structures^19^. The LBP-8 structure was solved in the C121 space group at high resolution (1.3 Å), with the asymmetric unit containing a monomer, which was consistent with size exclusion chromatography (Fig. 1B). Refinement and model statistics are summarized in Table 1. The crystal structure includes all 137 amino acids of wild type LBP-8, 92 waters, and two sulfate anions. LBP-8 adopts a typical lipocalin fold, present in all FABPs, consisting of a N-terminal alpha helix-turn-helix motif lid (αA- αB) and a twisted beta barrel containing ten antiparallel strands (βA-βJ) (Fig. 1A). The interior cavity is lined with polar and hydrophobic residues generating a solvent accessible surface area of 825 Å^2^ and volume of 1170 Å^3^ (Fig. 1C)^20^. There are fragments of continuous electron density present throughout the pocket; however, we were unable to model in a fatty acid with confidence. We attempted to co-crystallize LBP-8 with oleoylethanolamide (OEA), palmitic acid, and stearic acid, but all crystals yielded weak and fragmented density within the pocket. We do predict that fatty acid is binding to LBP-8 based on lipid MS data (Table 2), therefore, the fragmented electron density likely reflects that only a fraction of the LBP-8 in the crystal bound to fatty acid, or the fatty acid does not adopt a preferred conformation in the pocket. A network of eleven waters are present in the putative lipid binding pocket and anchored via hydrogen bonds with amino acids glutamine 56 and arginine 132.

**Table 1:** X-ray data collection and refinement statistics.

Data collection	LBP-8 Apo
Space group	C121
Cell dimensions	
*a*, *b*, *c* (Å)	46.9, 41.9, 70.9
α, β, γ (°)	90, 91.1, 90
Resolution (Å)	28.72–1.3 (1.347–1.3)
*R*_pim_	0.018 (0.274)
*I*/σ*I*	33.3 (1.6)
Completeness (%)	96.3 (67.9)
Redundancy	6.9 (4.8)
**Refinement**	
Resolution (Å)	1.3
No. reflections	32686 (2491)
*R*_work_/*R*_free_ (%)	19.04/21.01
No. atoms	
Protein	1142
Water	92
B-factors	
Protein	30.8
Ligand	37.6
Water	37.3
R.m.s. deviations	
Bond lengths (Å)	0.008
Bond angles (°)	0.9
Ramachandran favored (%)	100
Ramachandran outliers (%)	0
PDB accession code	6C1Z

Values in parenthesis indicate highest resolution shell (1.347–1.30 Å).

To identify conserved structural features between LBP-8 and other FABPs, we used the DALI server, which identifies similar protein structures based on root mean square deviations (r.m.s.d)^21^. This approach was critical since FABPs show low overall sequence conservation exemplified by the fact that the closest homolog by sequence is heart FABP at 49% similarity and 37% identity. Multiple FABP structures were found to be similar in 3D fold to LBP-8; however, we focused our analysis on the most similar structure that contained a bound fatty acid: FABP4 in complex with linoleic acid (PDB code 2Q9S)^17^. ProSMART ALIGN was used for alignment, superposition, and determining the structural conservation between the LBP-8 and FABP4 structure^22^. The main-chain dissimilarity scores were mapped onto the superposed structures with yellow depicting residues that have a similar local conformation, and gradually changing to red indicating comparative structural dissimilarity; white signifies unaligned residues (Fig. 1D). There are no major differences between the peptide backbones except for the loop between βG-βH, which is due to a three amino acids insertion in LBP-8. We additionally analyzed the structural conservation of side chains. Most side chain deviations were present in surface exposed residues, which is expected due to differential crystal packing and surface solvent interactions. The most divergent is an arginine side chain present in both structures (R81 in LBP-8 and R79 in FABP4). In FABP4, R79 is curled into the interior of the protein, where it can interact with D77 and solvent; however, in LBP-8, tyrosine 83 occupies this space, which positions R81 to the exterior surface.

### The lipid sensing portal region

**Figure 2:**
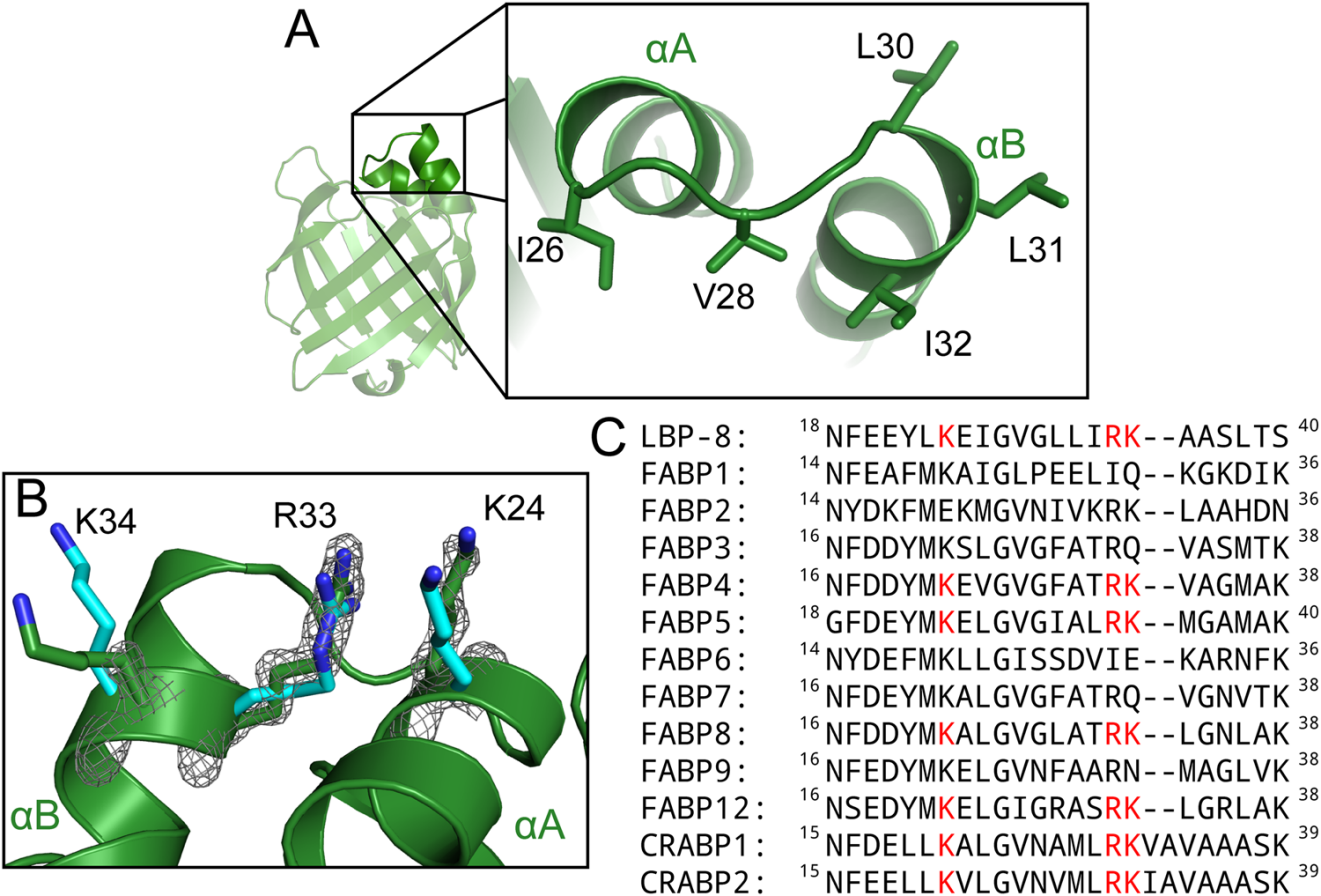
The portal region of LBP-8 contains a hydrophobic patch for interacting with membranes and conserved nuclear localization signal. (**A**) Zoomed in view of the LBP-8 (green) portal region with hydrophobic residues depicted as sticks. (**B**) Superposition of LBP-8 and FABP5 (PDB code 4LKT, cyan) with putative NLS residues depicted as sticks (C, green or cyan; N, blue). (**C**) Sequence alignment of LBP-8 with the human iLBPs. Residues that comprise the NLS are colored red.

The portal region of FABPs, which is comprised of a helix-turn-helix motif (αA- αB), plays a vital role in protein-membrane interactions, protein localization, and ligand sensing. Many FABPs, such as FABP2, FABP3, FABP4, and FABP7 have been described as “collisional” FABPs because they interact with the membrane via the alpha helical lid^23^. Positively charged residues within the αA and αB helices mediate electrostatic interactions with negatively charged phospholipid surfaces. Additionally, a hydrophobic patch within the turn region of the helix-turn-helix motif mediates insertion of the helical lid into the membrane^24,25^. The LBP-8 structure contains two lysines (K24 and K34) and an arginine (R33) present in the alpha helical lid, and a hydrophobic patch (25-IGVGLLI-32) within the turn region, suggesting LBP-8 is a “collisional” FABP, and directly interacts with membranes or membrane proteins to acquire fatty acids (Fig. 2A,B). LBP-8 was previously shown to localize to the lysosome; therefore, we predict LBP-8 utilizes the collisional mechanism to obtain fatty acid ligands from lysosomal membranes^8^.

The portal region of FABPs has also been reported to mediate nuclear localization. Though no nuclear localization sequence (NLS) is present in the primary sequence of FABPs, a three-dimensional structural NLS consisting of conserved lysines and an arginine was discovered in cellular retinoic acid binding protein 2 (CRABP-II), FABP4, and FABP5^15–17^. The NLS is stimulated through the binding of “activating” ligands, which stabilize the NLS, supporting interaction with nuclear importins^16^. LBP-8 was previously reported to localize to the nucleus in *C. elegans* upon overexpression of *Lipl-4*; therefore, we sought to determine if LBP-8 also contained a structural NLS^8^. We performed a structural alignment of the ten human FABPs and two human CRABPs, and found that LBP-8 contained the conserved NLS sequence, along with FABP4, FABP5, PMP2, FABP12, and both CRABPs (Fig. 2C). We then aligned our LBP-8 structure with a structure of FABP5 in complex with linoleic acid (PDB code 4LKT), which drives nuclear localization, and found the LBP-8 NLS residues (K24, R33, and K34) directly overlaid with the FABP5 NLS residues (Fig. 2B). This suggests that these positive residues were co-opted into a role to drive active nuclear translocation through interaction with importins for FABPs, and this mechanism is likely conserved in LBP-8. Indeed, deletion of residues containing the putative NLS ablated nuclear translocation^8^.

### LBP-8 binds to a range of fatty acids with preference for monounsaturated fatty acids

**Figure 3:**
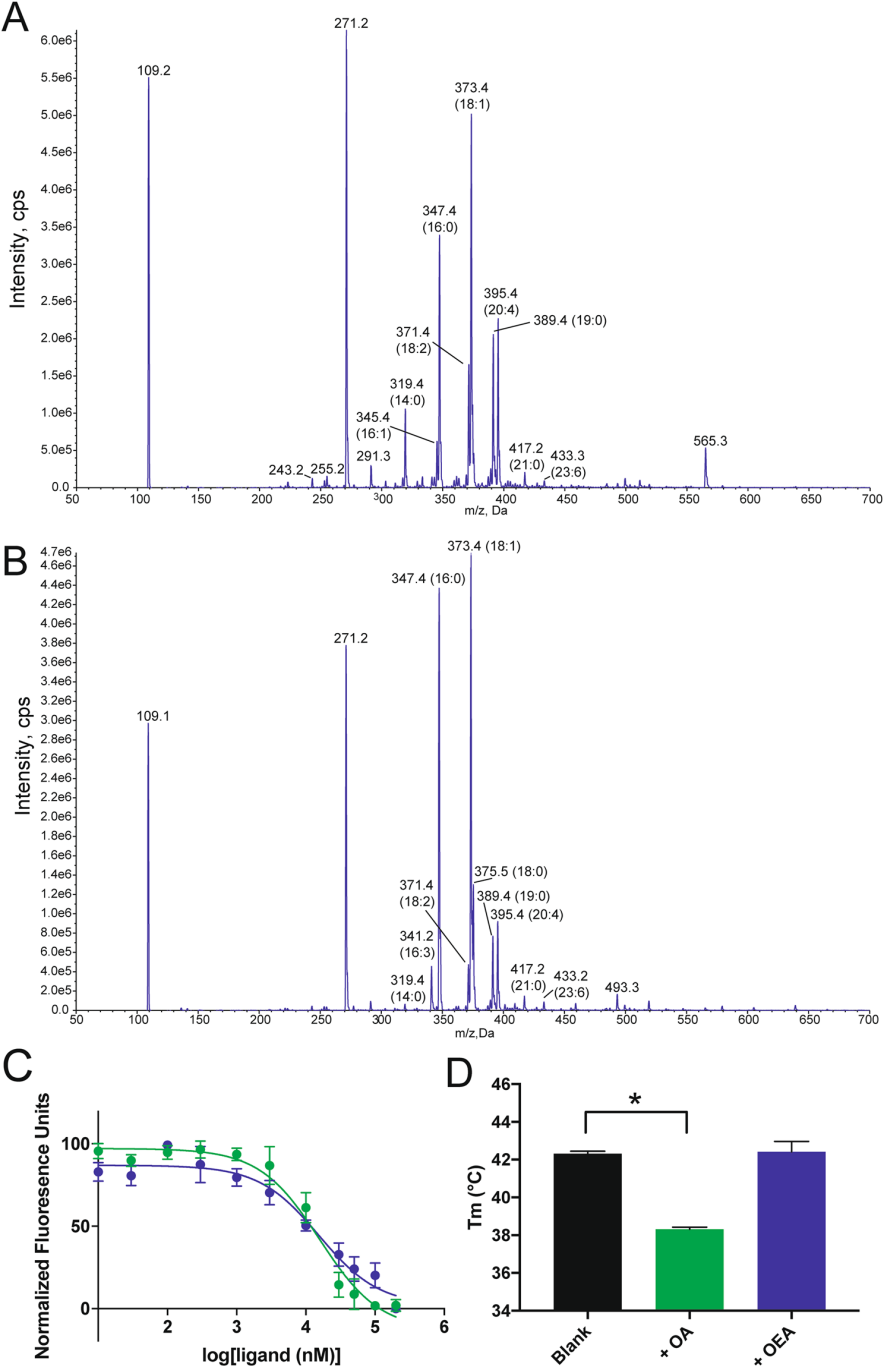
LBP-8 binds to a diverse array of saturated and unsaturated long-chained fatty acids. (**A**) Mass spectra (positive mode) of carboxyl group containing lipids extracted from LBP-8 incubated with *C. elegans* lipid extracts. (**B**) Mass spectra (positive mode) of carboxyl group containing lipids extracted from LBP-8 purified from *E. coli*. In both (**A,B**), peaks are identified using fatty acid nomenclature (fatty acyl length: number of double bonds). (**C**) Fluorescent ligand, 1,8-ANS, bound to LBP-8 was competed off with increasing amounts of oleic acid (green) and OEA (blue). Curves represent average of three independent replicates +/− SEM, conducted in triplicate, followed by normalization of curves. (**D**) Oleic acid (OA, green) decreased the thermal melting temperature of LBP-8 compared to no ligand (Blank) and OEA (blue). Each bar represents the average of three independent replicates +/− SEM, each conducted in triplicate. *p < 0.05 (significance was determined by one-way ANOVA followed by Dunnett’s multiple comparisons test).

Despite multiple attempts to crystallize LBP-8 in complex with fatty acids, only the apo-form of LBP-8 crystallized. Previously, we showed that LBP-8 bound to arachidonic acid (AA), ω-3 arachidonic acid (ω-3 AA), dihomo-γ-linoleic acid (DGLA), and oleoylethanolamide (OEA) in a dose dependent manner^8^. However, in order to identify all putative ligands, we took a discovery-based MS approach. Purified LBP-8 from *E. coli* was exposed to whole lipid extracts from *C. elegans*, re-purified through size-exclusion chromatography, and the bound fatty acids were identified through liquid chromatography/mass spectrometry (LC/MS) (Fig. 3A). Co-purified *E. coli* fatty acids were also determined by LC/MS and treated as background (Fig. 3B). To enhance signal and permit fatty acid quantification, we generated 3-picoylamide fatty acid derivatives, which selects for carboxyl containing lipids, and used precursor ion scan selecting for the loss of the 3-picoylamide ion^26^. Identification of the lipids from each experiment, and their relative percentages are recorded in Table 2. The major lipid species co-purified with LBP-8 from *E. coli* were palmitic acid (16:0) and oleic acid (18:1). Upon exposure to *C. elegans* lipid extracts, there was a shift in the binding preference of LBP-8. The relative amount of stearic acid (18:0) and palmitic acid (16:0) that co-purified with LBP-8 was greatly decreased, while there was an increase in the relative amount of myristic acid (14:0) and unsaturated fatty acids, such as arachadonic acid (20:4), linoleic acid (18:2), and palmitoleic acid (16:1). While the relative percentage of oleic acid decreased, it was still the most abundant fatty acid that bound to LBP-8. Additionally, two odd-chained fatty acids, heptadecanoic acid (17:0) and pentadecylic acid (15:0), co-purified with LBP-8 following exposure to *C. elegans* lipid extracts, which were not detectable in the LBP-8 purified from *E. coli*.

**Table 2:** Identification and relative quantification of lipids co-purified with LBP-8 via MS.

m/z (Da)	Adduct	Identified Lipid	Relative Percentage	
			*E. coli*	*C. elegans*
303.2	M + H	13:1	0.00%	0.48%
317.3	M + H	14:1	0.00%	0.74%
319.4	M + H	Myristic Acid (14:0)	0.47%	5.58%
333.4	M + H	Pentadecylic acid (15:0)	0.00%	0.78%
341.2	M + H	Hexadecatrienoic acid (16:3)	3.45%	0.85%
345.4	M + H	16:1	0.00%	3.56%
347.4	M + H	Palmitic Acid (16:0)	32.47%	19.44%
361.4	M + H	17:0	0.00%	0.92%
371.4	M + H	Linoleic Acid (18:2)	3.40%	9.27%
373.4	M + H	Oleic Acid (18:1)	35.72%	28.93%
375.5	M + H	Stearic Acid (18:0)	9.00%	2.29%
389.3	M + H	Nonadecylic Acid (19:0)	0.78%	1.52%
391.4	M + H	Hydroxy Stearic Acid	5.70%	11.24%
395.4	M + H	Arachadonic Acid (20:4)	6.82%	12.72%
417.2	M + H	Heneicosylic acid (21:0)	1.07%	1.14%
433.3	M + H	23:6	0.70%	0.54%
639.1	M + H	PS(20:3(8Z,11Z,14Z)/0:0)	0.44%	0.00%

The lipid MS analysis suggested that LBP-8 does not bind to one fatty acid selectively but is capable of binding many fatty acids. However, LBP-8 does have a preference for unsaturated fatty acids, such as oleic acid, when presented with a variety of lipids. Previously, oleoylethanolamide (OEA), a monounsaturated fatty amide, was shown to bind to LBP-8 with higher affinity compared to other unsaturated fatty acids like arachidonic acid and dihomo-γ-linoleic acid. Due to the high abundance of oleic acid that co-purified with LBP-8, we sought to compare the affinity of LBP-8 for OEA and oleic acid. A fluorescence-based ligand binding assay was used to compare the affinity of oleic acid and OEA, and both had very similar Ki’s, suggesting oleic acid, along with OEA, are high affinity ligands of LBP-8 (Fig. 3C).

To further analyze the effect of oleic acid binding to LBP-8, we conducted a thermal shift assay with LBP-8 in the presence of different ligands. To our surprise, oleic acid drastically destabilized LBP-8, decreasing the melting temperature (T_m_) by ~4 °C compared to apo, while OEA had no effect on the melting temperature (Fig. 3D). Ligands typically stabilize a protein upon binding, but there are instances when ligands destabilize a protein^27,28^. In this case, oleic acid selects for a less stable LBP-8 conformer.

### Analysis of the ligand binding pocket

To gain insight into ligand binding, we compared the LBP-8 interior binding pocket with other FABPs. As stated previously, the interior cavity of LBP-8 has a solvent accessible surface area of 825 Å^2^ and volume of 1170 Å^3^ (Fig. 1C)^20^. This interior cavity volume is similar to FABP9, smaller than FABP6 and FABP1, but larger than the other human FABPs (Table 3). While all FABPs bind medium to long-chained fatty acids, FABP6 and FABP1 bind to larger hydrophobic molecules such as bile acids, heme, and acyl-CoA^29,30^.

**Table 3:** Interior cavity surface area and volume of human FABPs and LBP-8.

Protein	PDB code	Surface Area (Å^2^)	Volume (Å^3^)
Apo-FABP6	5L8I	1069.6	1482.8
FABP1	3STK	978.1	1429.2
**LBP-8**	**6C1Z**	**825**	**1170.3**
FABP7	5URA	770.6	1086.1
FABP3	5B27	768.1	1034.2
Apo-FABP2	1IFB	707.8	971.7
Apo-FABP9	4A60	703	1170.3
FABP8	3NR3	684.4	941.6
Apo-FABP5	4LKP	664.1	916.1
Apo-FABP4	3Q6L	636.5	936.6

**Figure 4:**
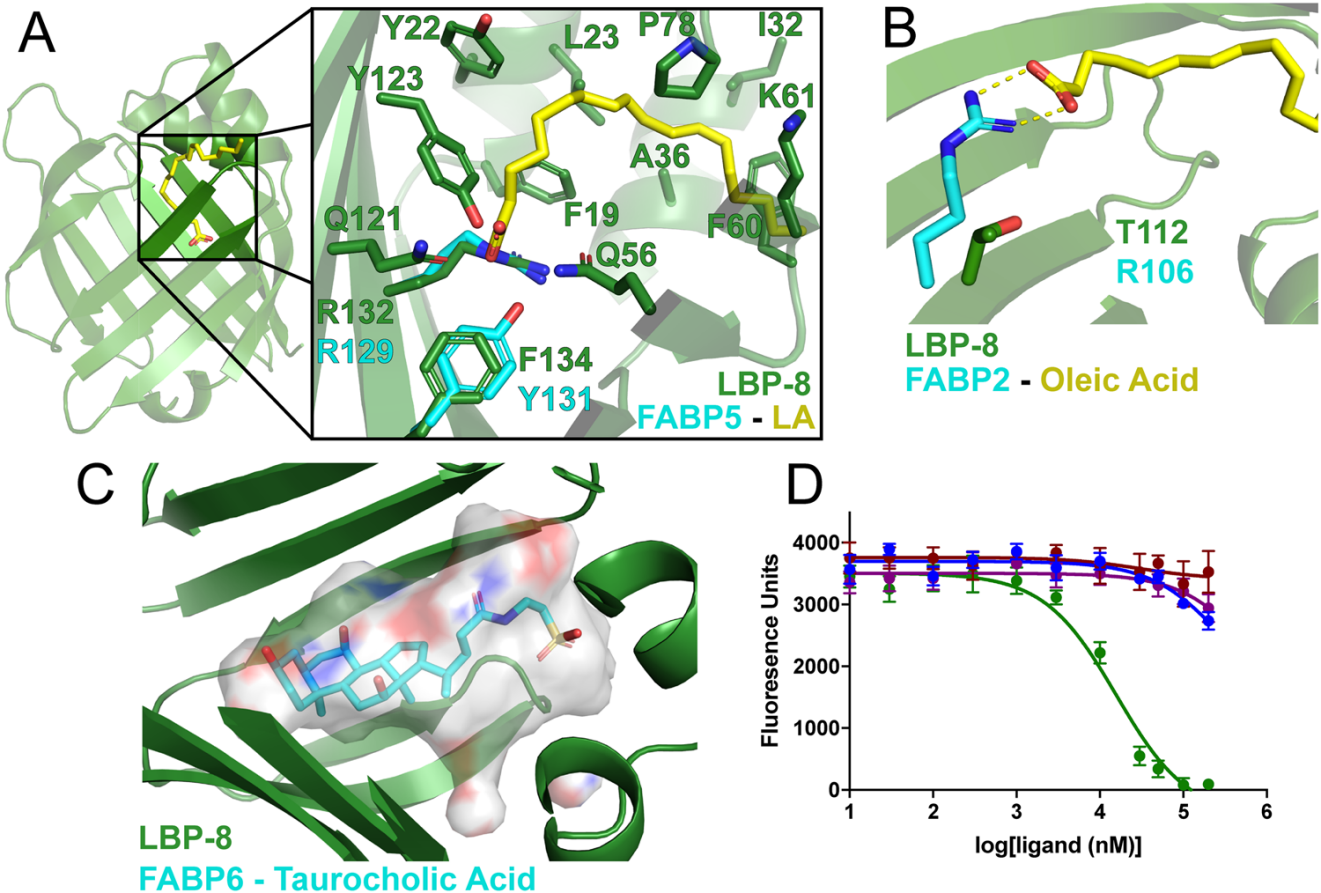
Comparison of ligand binding pocket of LBP-8 with FABPs. (**A**) LBP-8 (green) is aligned with a structure of FABP5 (PDB code 4LKT, cyan) bound to linoleic acid (yellow). LBP-8 residues 3.5 Å away from linoleic acid are displayed. LBP-8 contains the conserved R132 that is also present in FABP5, R129, which electrostatically interacts with the head group of linoleic acid. FABP5 also contains a highly conserved tyrosine, Y131, which hydrogen bonds with the head group of linoleic acid, while LBP-8 contains a phenylalanine, F134. (**B**) LBP-8 (green) is aligned with FABP2 (PDB code 2MO5, cyan) bound to oleic acid (yellow). The arginine, R106, electrostatically interacts with the head group of oleic acid, but LBP-8 contains a threonine, T112, at this residue. (**C**) LBP-8 (green) is aligned with a structure of FABP6 in complex with taurocholic acid (PDB 1O1V, cyan). The solvent accessible surface of LBP-8’s interior pocket is displayed in transparent white with charged surfaces colored red (negative) and blue (positive) around the ligand. (**D**) Fluorescent ligand, 1,8-ANS, bound to LBP-8 was competed off with increasing amounts of oleic acid (green), cholic acid (blue). Taurocholic acid (red), and glycocholic acid (purple). Curves represent average of three independent replicates +/− SEM, conducted in triplicate.

Next, we compared the interior cavity side chains of LBP-8 with the other FABPs. The interior cavity of LBP-8 is lined with hydrophobic residues (F19, F60, L65, F67, F73, F94, F110, T112, and F134), which can stabilize fatty acyl tails of fatty acids via hydrophobic interactions. This is a trait found throughout the lipocalin family, with F19, F60, F67, and F73 being highly conserved residues. Additionally, the interior cavity is lined with several polar residues (Q56, Q121, Y123, and R132), which are capable of interacting with charged head groups of fatty acids via hydrogen bonding (Fig. 4A). Arginine 132 is highly conserved and is present in all human and *C. elegans* FABP isoforms; it has been shown to participate in electrostatic interactions with the head group of the bound fatty acid in many holo-FABP structures (Fig. 4A)^15,19,31^. The other interior polar residues in LBP-8 are not well conserved in human or *C. elegans* FABPs, suggesting R132 is likely an important residue for mediating lipid binding throughout the FABP family.

On the other hand, several amino acids that are highly conserved in other lipocalin family of proteins are not present in LBP-8. For instance, most *C. elegans* and human FABPs, excluding FABP1, FABP2, and FABP6, contain a tyrosine that is two residues downstream of the conserved arginine 132 (LBP-8 numbering), which also mediates electrostatic interactions with the head group of bound fatty acids (Fig. 4A)^11^. LBP-8 contains a phenylalanine (F134) at this position, which would disrupt the electrostatic interactions with the head group (Fig. 4A). Since FABP1, FABP2, and FABP6 also lack this tyrosine, we further compared LBP-8 to these FABPs, which are all capable of binding to hydrophobic molecules other than just medium to long chain fatty acids^29,30^. A structure of the FABP2-oleic acid complex shows that the head group of oleic acid interacts with arginine 112 (LBP-8 numbering), resulting in fatty acid bound deeper in the pocket compared to other holo-FABP structures (Fig. 4B). Although this residue is present in all FABPs except FABP1 and FABP6, the fatty acid only adopts this deep pocket conformation in FABP2. This conformation possibly occurs because of the absence of the conserved Y134 (LBP-8 numbering) at the C-terminus, leading to a new interaction site at R112 (LBP-8 numbering). However, LBP-8 has a threonine (T112) at this residue, like FABP1, rather than an arginine, which would not recapitulate the electrostatic interaction as seen in the FABP2-oleic acid structure (Fig. 4B). Similarly, FABP6 contains a serine at this residue.

Given LBP-8’s pocket size and composition, we hypothesize that LBP-8 would bind a more diverse set of lipids similar to FABP1 and FABP6. In supporting this hypothesis, our lipid MS analysis identified a wide variety of lipids co-purified with LBP-8, including large lipids such as a phosphatidylserine species and a 23-carbon fatty acid, which have never been identified to bind to FABPs before now (Table 2). In order for the binding pocket to accommodate these lipids, we expect an opening of the portal region, enlarging the interior cavity, as seen in previous FABP structures^15^. To be noted, derivatization of lipids proceeding LC/MS prevented detection of non-carboxyl-containing lipids. Thus, there might be more lipids bound to LBP-8.

Bile acids such as cholic acid, taurocholic acid, and glycocholic acid are ligands for FABP6 and were significantly reduced in *lipl-4* transgenic worms that had extended lifespan in an LBP-8 dependent manner^8,32^. Therefore, we postulated that these bile acids bound to LBP-8. We aligned a structure of FABP6 in complex with taurocholic acid with LBP-8 to determine if the LBP-8 pocket would accommodate bile acid binding. The ligand fit nicely within the solvent accessible surface of the pocket with only a few minor steric clashes (Fig. 4C). We then performed a fluorescence-based ligand binding assay with LBP-8 and cholic acid, taurocholic acid, and glycocholic acid. Compared with oleic acid, none of the bile acids bound or bound with very low affinity (Fig. 4D). Further experimentation is required to determine if LBP-8 is capable of binding to larger lipid molecules, should these ligands be determined biologically relevant.

### Mutational analysis of LBP-8 ligand binding pocket

Given the LBP-8 structure, we hypothesized that polar residues Q56, Q121, Y123, and R132 lining the interior cavity of LBP-8 stabilize the head group of fatty acids to mediate ligand binding (Fig. 4A). Though Q56, Q121 and Y123 are not conserved in human FABPs, they are apposed to the carboxyl head group of many fatty acids structurally aligned with LBP-8. Therefore, we created several mutational constructs that contained various combinations of these residues mutated to alanine. All constructs were purified successfully and eluted at the same volume as wild-type LBP-8 in size exclusion chromatography.

**Figure 5:**
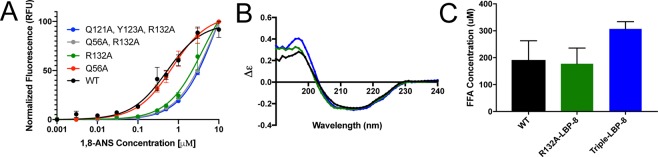
Analysis of ligand binding pocket mutants. A. Fluorescent probe, 1,8-ANS, was titrated into wild-type LBP-8 and mutant constructs. Curves depict average of experiment performed in triplicate +/− SEM, followed by normalization of curves. Mutant constructs with R132A mutant have significantly reduced affinity for 1,8-ANS. (**B**) Circular dichroism spectra in molar extinction units (Δε) for WT-LBP-8 (black), R132A-LBP-8 (green), and Triple-LBP8 (blue) mutants. (**C**) Average amount of fatty acid bound to 500 μM WT-LBP-8, R132A-LBP-8 and Triple-LBP-8 mutants. Each bar represents the average of three independent replicates +/− SEM.

In order to test the impact of these residues on fatty acid binding, we first attempted to use a fluorescence-based competition assay, but many of our LBP-8 mutants had a significantly reduced binding to the fluorescent probe (1,8-ANS), preventing us from accurately comparing fatty acid binding to wild-type LBP-8 (Fig. 5A). Mutating asparagine 56 did not alter the affinity of the probe for LBP-8 but mutating the highly conserved arginine 132 significantly reduced probe affinity. All other constructs that included this arginine 132 mutant exhibited significantly lower affinity for probe. This led us to hypothesize that arginine 132 plays an essential role in fatty acid binding.

Since our fluorescent-based competition assay was insufficient to directly compare fatty acid binding between our constructs, we used another technique to probe fatty acid binding. Moving forward, we only utilized the R132A (R132A-LBP-8) and Q121A, Y123A, R132A (Triple-LBP-8) mutant constructs, since Q56 appeared to play an insignificant role in binding. We verified proper folding of these constructs with circular dichroism, which revealed no difference between the wild-type and mutant proteins (Fig. 5B). We then used a coupled enzymatic reaction and colorimetric probe, to compare the total amount of fatty acid bound to wild type and mutant proteins when purified from *E. coli*. The R132A-LBP-8 protein had a similar amount of fatty acid bound compared to wild type protein. Surprisingly, the Triple-LBP-8 protein bound to a greater amount of fatty acids compared to wild type (Fig. 5C). Though our mutants had reduced affinity for 1,8-ANS, they actually bound to more fatty acid, suggesting the mechanism of binding for these ligands differ. These surprising results could be due to the generation of a larger and more hydrophobic pocket in our mutants, which would accommodate more fatty acid, yet disrupt 1,8-ANS binding.

## Discussion

As a very conserved family of lipid binding proteins, FABPs share similarities in structure and fold, but there is vast diversity in sequence, ligand specificity, and function within the family. Much effort has been directed towards understanding the biology of human FABPs and we have high-resolution structures of all human FABPs, with the exception of FABP12. Yet, little is known about FABPs in other organisms, which limits our understanding on the evolution of these proteins. Here, we have expanded our knowledge of the FABP family by reporting the first structure of a *C. elegans* FABP, LBP-8, and exploring its ligand specificity.

Though LBP-8 has little sequence similarity with human FABPs, it shares many of the same structural motifs. It contains a portal region similar to other FABPs, harboring a structural NLS present in many other FABPs. Additionally, LBP-8 binds to similar types of hydrophobic molecules known to bind to all FABPs, showing preference for long chained fatty acids. These congruent features support the conservation of FABP proteins on the structural basis, and the translatability of FABP biology across different species.

On the other hand, our studies also discover differences present in LBP-8 compared with human FABPs, many of which are found in the interior cavity. First, though LBP-8 contains a conserved arginine 132, it lacks a highly conserved tyrosine two residues downstream, which makes up what has been termed the P2 motif (Arg – x – Tyr) that is responsible for stabilizing the head group of bound fatty acids^11,33,34^. Additionally, LBP-8 lacks a conserved arginine at residue 112 that stabilizes the head group of fatty acids in FABP2. The absence of these conserved amino acids and having large pocket volume suggest that fatty acids can bind variably, possibly explaining why there was such diversity in our lipid MS data and disordered electron density within the interior of the crystal structure.

We attempted to ablate fatty acid binding through mutating conserved polar amino acids within the interior pocket, namely arginine 132, glutamine 121 and tyrosine 123, which we predicted to electrostatically interact with the carboxyl head group of fatty acids. To our surprise, mutating these residues did not reduce fatty acid binding, but rather increased binding. This suggests that though these conserved amino acids may play a role in orienting the carboxyl head group in the pocket, they do not drive fatty acid binding. A similar increase in ligand affinity, 30-fold, was discovered in human FABP2 upon mutating arginine 106 to an alanine, which disrupted the electrostatic interaction between the basic residue and the carboxyl head group of oleic acid^35^. Though the enthalpy of binding was decreased upon loss of the electrostatic interaction, this was more than compensated with an increase in entropy^35^. Our data concurs with this previous study which showed that binding to fatty acid is driven more entropically than enthalpically. The hydrogen bonding that occurs between a fatty acid head group and polar residues within the pocket are not necessary for binding. Hydrophobic effects appear to have a greater impact on binding than these hydrogen bonds. However, 1,8-ANS binds to LBP-8 through a very different mechanism than fatty acids. A previous crystal structure of human FABP3 bound to 1,8-ANS showed hydrogen bonding between the sulfonic acid group of the ligand and a water network coordinated by the highly conserved arginine 126 (R132 LBP-8 numbering)^36^. This explains why mutating arginine 132 in LBP-8 significantly reduced 1,8-ANS binding. Given this data, we suspect that entropy is the main driver in fatty acid binding, while enthalpy is the main driver of 1,8-ANS binding to LBP-8.

Our lipid MS analysis showed that LBP-8 bound to a diverse array of fatty acids. A low degree of lipid selectivity is a common trait found throughout the FABP family^37^. However, while FABPs are capable of binding an array of hydrophobic molecules, they have evolved to selectively respond through conformational dynamics to a few lipids. For instance, polyunsaturated fatty acids binding to FABP5 activate its localization to the nucleus and the up-regulation of PPARβ/δ target genes, while saturated fatty acids binding does not activate these FABP5 functions^15^. Activating ligands allosterically communicate with an “activation switch”, which is two hydrophobic residues that lie at the interface between the α2 helix of the portal region and the β2 loop (M35 and L60). Polyunsaturated fatty acids stabilize this switch, which stabilizes the NLS, stimulating nuclear localization^15^. Similarly, a PPARα agonist, GW7647, altered the conformation of residues on loops adjacent to the portal region of FABP1, which promoted interaction with PPARα and PPARα transactivation^38^. We hypothesize that the LBP-8 portal region and the surrounding loops mediate a similar ligand-controlled activation switch. In support of this, LBP-8 also contains hydrophobic residues (A35 and F60) at the same activation switch region found in FABP5. Despite the fact that many fatty acids are capable of binding to LBP-8, only select fatty acids may stimulate the life-extending effects of LBP-8. Consistently, although LBP-8 is capable of binding to saturated fatty acids, polyunsaturated fatty acids, monounsaturated fatty amide OEA, and monounsaturated fatty acid oleic acid, only OEA and oleic acid are shown to prolong *C. elegans* lifespan so far^8,39^.

Oleic acid plays a key role in many cellular events including remediation of inflammation, stimulation of lipid metabolism, and increased insulin sensitivity, yet the mechanisms by which oleic acid mediates all these effects aren’t fully understood^40–42^. In this study, we showed LBP-8 co-purified with oleic acid, a monounsaturated fatty acid, from *C. elegans* lipid extracts. Additionally, oleic acid bound to LBP-8 with similar affinity as OEA, suggesting LBP-8 prefers monounsaturated fatty acyls. We propose LBP-8 is the main monounsaturated fatty acyl transporter to the nucleus to regulate aging.

## Materials and Methods

### Materials and reagents

Chemicals were purchased from Sigma, Fisher or Acros Organics. The vector for His-tagged tobacco etch virus (TEV) was a gift from John Tesmer (University of Texas at Austin). The pMCSG7 (LIC_HIS) vector was provided by John Sondek (University of North Carolina at Chapel Hill). DNA oligonucleotide primers were synthesized by IDT (Coralville, IA).

### Cloning and mutagenesis

Full-length, wild-type *Caenorhabditis elegans* LBP-8 (residues 1–137) from was subcloned into pMCSG7-His vector. The NLS-deficient mutant (LBP-8 NLSm: K24A, R33A, and K34A) and lipid binding deficient mutants (combinations of Q121A, Y123A, and R132A) were generated in pMCSG7-His. All mutagenesis was accomplished using the megaprimer method^43^.

### Protein expression and purification

Full-length *Caenorhabditis elegans* LBP-8 in the pMCSG7 vector (wild-type and mutants) was transformed into *Escherichia coli* strain BL21 (DE3) cells and expressed as a His_6_ fusion containing a tobacco etch virus protease cleavage site to facilitate tag removal. Cultures (1 liters in TB) were grown to an *A*_600_ of ~0.6 and induced with 0.5 mM isopropyl β-d-1-thiogalactopyranoside at 22 °C for ~18 hours. Cell mass was harvested, lysed through sonication in a buffer containing 20 mM Tris HCl pH 7.4, 150 mM NaCl, 25 mM imidazole, 5% glycerol, lysozyme, Dnase A, and 100 uM phenylmethylsulfonyl fluoride. LBP-8 was purified by nickel affinity chromatography and the His tag was cleaved by tobacco etch virus protease at 4 °C overnight with simultaneous dialysis into a buffer containing 20 mM Tris HCl pH 7.4, 150 mM NaCl, and 5% glycerol. Cleaved LBP-8 was purified from His tag through nickel affinity chromatography followed by gel filtration chromatography using a HiLoad 16/60 Superdex 75 column. For ligand binding studies, LBP-8 was unfolded and refolded to remove bound *E. coli* lipids. To do so, LBP-8 sequestered in inclusion bodies was solubilized and unfolded by resuspension and sonication in denaturing buffer (20 mM Tris HCl pH 8.0, 300 mM NaCl, 8 M urea, 5 mM 2-mercaptoethanol, and 25 mM imidazole). Unfolded LBP-8 was refolded on a 5 mL HisTrap FF affinity column using a linear gradient to remove urea, and then eluted using imidazole. Refolded LBP-8 was further purified by gel filtration chromatography using a HiLoad 16/60 Superdex 75 column.

### Crystallization, data collection, structural refinement

Pure wild-type, full-length LBP-8 was concentrated to 15 mg mL^−1^ in 20 mM Tris HCl pH 7.4, 150 mM NaCl, and 5% glycerol. Crystals of LBP-8 were grown over two weeks via sitting drop vapor diffusion at 4 °C from solutions containing 1 μL LBP-8, 1 μL mother liquor (2.81 M ammonium sulfate and 0.25 M potassium formate), and 0.7 μL LBP-8 seed stock. Crystals were cryoprotected by immersion in 2 M ammonium sulfate, 0.325 M potassium formate, and 20% glycerol and flash frozen with liquid nitrogen. Data were collected remotely from the Southeast Regional Collaborative Access Team at the Advanced Photon Source, 22ID beamline (Argonne National Laboratories, Chicago, IL). Data were processed and scaled using HKL-2000 (HKL Research, Inc., Charlottesville, VA)^44^ and phased by molecular replacement using Phaser-MR (Phenix, Berkeley, CA)^45^. The structure was phased using a previously solved crystal structure of human FABP3 (3WVM) as a search model^19^. Structure refinement and validation was performed using PHENIX (Phenix, Berkeley, CA) (version 1.11.1), and model building was performed in COOT (MRC Laboratory of Molecular Biology, Cambridge, UK)^45,46^. PyMOL (version 1.8.2; Schrödinger, New York, NY) was used to visualize structures and generate figures.

### LBP-8 lipid exchange with *C. elegans* lipids

A synchronous population of approximately 500,000 day 1, N2 worms were grown at 20 °C on OP50. Worms were washed 3x in PBS, frozen into small pellets in liquid Nitrogen, and stored at −80 °C. The worms were later cracked using a Cellcrusher. The cracked worms were then ground using a pestle and mortar, which had been chilled with liquid Nitrogen, until no intact worms remained. Liquid nitrogen was added to the sample in both the Cellcrusher and pestle and mortar as needed to maintain a cold temperature. Lipids were extracted from the *C. elegans* lysates using the Bligh and Dyer method^47^. Briefly, 1.6 grams of homogenized *C. elegans* lysates was resuspended in 5 ml methanol and 2.5 ml chloroform and vortexed for 30 minutes. Undissolved material was removed, followed by the addition of 2.5 ml 0.1 M NaCl. Additional methanol and chloroform were added to separate the aqueous and organic phase. The organic phase was collected and dried with nitrogen gas. Dried lipid extracts were resuspended in LBP-8 sizing buffer (20 mM Tris HCl pH 7.4, 150 mM NaCl, and 5% glycerol) plus 0.5% DMSO, sonicated for 15 minutes, and rocked at 4 °C overnight to form lipid vesicles. The lipid vesicles were incubated with purified LBP-8 at 4 °C overnight while rocking. Nonspecifically bound lipids were removed through gel filtration chromatography using a HiLoad 16/60 Superdex 75 column.

### Lipid derivatization and mass spectrometry

Lipids were extracted from LBP-8 purified from *E. coli* before and after exchange with *C. elegans* lipid extracts using the Bligh and Dyer method as described above^47^. Fatty acid derivatives were generated as previously described here^26^. Briefly, dried lipid extracts were incubated with 200 *μ*L of oxalyl chloride (2 M in dichloromethane) at 65 °C for 5 minutes, and then dried down with nitrogen gas. Then, 3-picolylamide fatty acid derivatives were formed through incubation with 3-picolylamine at room temperature for 5 minutes and then dried down with nitrogen gas. The fatty acid derivatives were resuspended in a 1:1 methanol: water solution for mass spec analysis. The sample was directly injected into the ABSciex QTRAP5500 mass spectrometer. Data was collected in positive-ion mode using a precursor ion scan selected for the precursor ion of picolylamine (109 *m/z*). Data was acquired and analyzed using LipidSearch software.

### Circular dichroism

Wild-type and mutant forms of LBP-8 were concentrated to ~0.8 mg/ml in 20 mM Tris HCl pH 7.4, 150 mM NaCl, and 5% glycerol. Circular dichroism (CD) studies were performed on a Jasco J-810 spectropolarimeter with a 1 mm cell. Wavelength scans measuring ellipticity signal were performed at 25 °C from 190 to 300 nm at intervals of 0.2 nm. Each scan is the average of three independent spectral scans. Ellipticity degrees were converted to molar extinction to account for slight variations in protein concentration. The α-helix/β-sheet ratios were calculated using the k2d3 server k2d3.ogic.ca/^48^.

### Competitive fluorescence-based binding assay

Quantification of ligand binding was conducted via competition with the probe 1-anilinonaphthalene-8-sulfonic acid (1,8-ANS), a small molecule whose fluorescence increases drastically when surrounded by a hydrophobic environment and which has been shown to bind an array of iLBPs with varying affinity^49^. Briefly, binding of 1,8-ANS was carried out in PBS (137 mM NaCl, 2.7 mM KCl, 10 mM Na_2_HPO_4_, 2 mM KH_2_PO_4_, pH = 8.0) in the presence of 250 nM LBP-8 that was unfolded and refolded to remove *E. coli* lipids and increasing amounts of fluorescent probe (0–30 µM). Blank measurements containing 1,8-ANS only were subtracted from each probe concentration tested, and the resulting fluorescent values were fit with a One-Site binding curve to determine the binding constant, K_D_. Competition assays were then carried out in the same buffer system using a constant concentration of 250 nM protein and 800 nM 1,8-ANS, with ligand added via 100X ethanol stocks to maintain an ethanol concentration of 1%. Following a one-hour incubation at 37 °C, data were collected on a BioTek Synergy NEO plate reader using an excitation wavelength of 360 nm and an emission wavelength of 525 nm. Blank wells containing only ligand and 1,8-ANS were subtracted from wells with protein at each ligand concentration tested. Data was processed in GraphPad Prism 7. All curves are the average of three independent experiments.

### Differential scanning fluorimetry (DSF)

Purified LBP-8 protein (7 μM) that was unfolded then refolded to remove bound *E. coli* lipids was incubated for 30 minutes with 20 μM of oleic acid, cholic acid, or OEA at room temperature. Lipid ligands were dissolved in ethanol and diluted in water so that the percentage of ethanol was held at 1% in the final reaction. SYPRO orange dye (Invitrogen) was then added at a 1:2000 dilution. Reactions were heated at a rate of 0.5 °C per minute, using a StepOne Plus Real Time PCR System (ThermoFisher). Fluorescence was recorded at every degree using the ROX filter (602 nm). Data were analyzed by first subtracting baseline fluorescence (ligands + SYPRO with no protein) and then fitting the curves using the Bolzman equation (GraphPad Prism, v6) to determine the Tm. One-way ANOVA was used to compare Tm’s of different ligands.

### Fatty acid quantification

Lipids were extracted and dried down with nitrogen gas from equal amounts of purified WT and mutant forms of LBP-8 using the Bligh and Dyer method as described above^47^. The dried lipid extracts were resuspended in PBS (137 mM NaCl, 2.7 mM KCl, 10 mM Na_2_HPO_4_, 2 mM KH_2_PO_4_, pH = 8.0). The total amount of fatty acid for each sample was determined using the Free Fatty Acid Assay Kit (Colorimetric), Cell Biolabs, San Diego, CA, USA. Data was analyzed in GraphPad Prism 7. All data represents the average of three replicates.

## Data Availability

The LBP-8 crystal structure dataset is available at the Protein Data Bank with the accession code 6C1Z. All other datasets generated and analyzed during the current study are available from the corresponding author on reasonable request.
